# Assessment of Venous Thromboembolism Awareness Among Surgical Ward Patients in Makkah, Saudi Arabia: A Cross-Sectional Study

**DOI:** 10.7759/cureus.27897

**Published:** 2022-08-11

**Authors:** Mohammed S Bosaeed, Rafal N Balubaid, Abdullah R Alharbi, Omar S Alhothali, Aseel K Haji, Hanan E Alkaabi, Renad A Miyajan

**Affiliations:** 1 Vascular and Endovascular Surgery, Al-Noor Specialist Hospital, Makkah, SAU; 2 Internal Medicine, College of Medicine and Surgery, Umm Al-Qura University, Makkah, SAU; 3 Medical School, College of Medicine and Surgery, Umm Al-Qura University, Makkah, SAU

**Keywords:** patient health awareness, safety patient, pulmonary embolism (pe), deep vein thrombosis (dvt), venous thromboembolism (vte)

## Abstract

Introduction

Venous thromboembolism (VTE) is the leading source of morbidity and mortality among hospitalized patients in Saudi Arabia. Currently, there is no literature on VTE knowledge and awareness among hospitalized patients in Saudi Arabia’s western region. Consequently, this study aimed to investigate the hospitalized patients’ awareness and perceptions of VTE and associated thromboprophylaxis in surgical wards in Makkah, Saudi Arabia.

Methods

A descriptive cross-sectional study was conducted on 301 patients who were admitted to the surgical ward in the Al-Noor Hospital for more than three days, between September and November 2021.

Results

The study found that patients who had higher education levels, and who were currently or previously receiving pharmacological/non-pharmacological thromboprophylaxis had a significantly higher knowledge score regarding VTE (p = < 0.05). On the other hand, a non-significant relationship was found between knowledge scores and age, gender, the reason for admission, and personal or family history of VTE (p = > 0.05). Spearman’s correlation analysis also revealed a highly significant positive correlation between the patients’ knowledge and attitude scores (r=0.21, p=<0.001).

Conclusion

This study revealed a lack of awareness among hospitalized patients about VTE, clinical presentation, and risk factors. Therefore, we encourage health care providers to educate patients about them.

## Introduction

Venous thromboembolism (VTE), commonly referred to as deep vein thrombosis (DVT), is caused by blood clots forming in the veins and can be fatal if it is accompanied by pulmonary embolism (PE). It is the third-leading cause of cardiovascular disease and the leading source of morbidity and mortality among hospitalized patients worldwide [1]. Annual VTE incidence rates in Europe are projected to range from 104-183 per 100,000 people [2]. Considering similar international rates, the actual number of VTE incidences in Saudi Arabia is unknown; however, it affects roughly 25,000 people every year [3]. The most common symptoms of PE are chest discomfort and shortness of breath [4]. Venous stasis, blood hypercoagulability, and endothelial damage (Virchow’s triad) are all risk factors for VTE [5].

Approximately 50% to 60% of the disease burden from VTE is associated with recent hospitalization [6]. Increasing patient awareness of VTE prevention and the need for thromboprophylaxis will likely boost their willingness to participate in VTE management through early mobilization and calf-pump exercises [7]. However, there is still a lack of public understanding about VTE. Only 28% of respondents in a poll of six developing nations were aware of PE symptoms, and only 19% were aware of DVT symptoms [6].

Various other studies have been carried out all over the world to assess the level of awareness and understanding about VTE among hospitalized patients. A 2007 study that looked into patient knowledge of VTE suggested that hospitalized patients should be given more information to help them prevent and recognize VTE [7]. A cross-sectional study was conducted in Riyadh, Saudi Arabia between 2015 and 2016 to assess patients’ awareness of VTE and satisfaction. The supplied related information found that hospitalized patients had an inadequate understanding of DVT and PE [8]. A study conducted in Jordan in 2019 on patients who received thromboprophylaxis yielded similar outcomes, corroborating the earlier findings [9].

There is currently no literature on VTE knowledge and awareness among hospitalized patients in Saudi Arabia’s western region. Consequently, the current study sought to investigate the hospitalized patients’ awareness and perceptions of VTE and associated thromboprophylaxis in surgical wards within Makkah, Saudi Arabia.

## Materials and methods

Study design, setting, and time frame

A descriptive cross-sectional study was carried out in Al-Noor Hospital, Makkah, Saudi Arabia, between September 2021 and November 2021.

Sample size

The study sought to estimate the awareness and perception of VTE and thromboprophylaxis among hospitalized patients in Makkah. The average number of patients who receive thromboprophylaxis in one month at Al-Noor Hospital is 942. The participants were selected via convenience sampling. With a confidence level of 95%, a margin of error of 5%, and a response distribution of 50%, the minimum recommended sample size for our study was determined to be 274.

Study population

Patients admitted to the surgical ward of Al-Noor Hospital in Makkah, Saudi Arabia, between September 2021 and November 2021 were included. The inclusion criteria included patients who were admitted to the surgical ward, over 18 years of age, and who were hospitalized for more than 72 hours. Exclusion criteria were critically ill patients, patients receiving ambulatory care or patients with cognitive impairment.

Data collection

Data were collected through a previously validated questionnaire in Arabic. The questionnaire was taken from a study conducted in Riyadh, Saudi Arabia [8]. Permission was obtained from the author of the study. We reviewed the questionnaire and found it suitable for our objective and population. The questionnaire is made up of three sections. The first included items about demographic data and family or personal history of VTE and thromboprophylaxis. The second included items to assess knowledge about PE and DVT, including symptoms, causes, risk factors, and prevention. The third section included items to assess the patients’ attitude on the perceptions of pharmacologic thromboprophylaxis, information received on VTE, level of satisfaction about receiving pharmacological venous thromboembolism prophylaxis, and the explanation that preceded it.

For knowledge items, every right answer was assigned a score of "1" and every wrong one, a score of "0". The respondent's level of knowledge about venous thromboembolism was defined as good if the study participant correctly responded to more than or equal to 80% of the knowledge assessment items; conversely, if their score was less than 80%, their knowledge was considered poor [8]. As for the participants’ attitudes, "strongly agree" and "agree" answers were assigned a score of "1" and "neutral", "disagree", and "strongly disagree" answers were assigned a score of "0".

The sample was classified into two groups depending on the their level of satisfaction. They were categorized as being "satisfied" if their level of satisfaction was equal to or above 80%, and "unsatisfied" if below 80% [8].

Ethical considerations

Ethical approval for the study was obtained from the institutional review board (IRB) of the Al-Noor Hospital, Makkah, Saudi Arabia. To ensure that all survey items were clear and comprehensive, the participants were interviewed by one of the researchers. A full description of the study and its objectives was provided to patients before asking them to participate in the study. Patients were interviewed after the third day of admission to ensure that they had the chance to receive any kind of information about VTE or thromboprophylaxis.

Statistical analysis

SPSS Statistics v. 26 (IBM Corp, Armonk, NY) was used for statistical analysis. Data were summarized by standard descriptive summaries: means and standard deviations (mean ± SD) for continuous variables, numbers, and percentages for categorical variables. A chi-squared test (χ2) was used for categorical values. Comparison between quantitative non-parametric variables was conducted using the Mann-Whitney U and Kruskal Wallis tests. Correlation analysis was performed using Spearman’s test. A p-value of less than 0.05 was considered statistically significant.

## Results

Table 1 shows that 41.6% of the studied patients were in the 31-50 age range, 61.8% were males, and 33.9% were university educated. In addition, 95.7% of the subjects were admitted for surgical purposes, 9% and 20.3% had a personal or family history of VTE, respectively. About 66% of the patients (66.1%) were currently receiving pharmacological/non-pharmacological thromboprophylaxis and 25.2% had a history of receiving them.

**Table 1:** Distribution of studied patients according to their demographic characteristics, reason for admission, and clinical history (N = 301)

Variable		No. (%)
Age Range (years)	18–30	55 (18.3)
	31–50	125 (41.6)
	51–70	85 (28.2)
	71+	36 (12)
Gender	Female	115 (38.2)
	Male	186 (61.8)
Education Level	Uneducated	40 (13.3)
	Less than high school	63 (20.9)
	High school	89 (29.6)
	Undergraduate	102 (33.9)
	Postgraduate	7 (2.3)
Reason for Admission	Chemotherapy	1 (0.3)
	Oncology (nonsurgical)	12 (4)
	Surgical	288 (95.7)
Personal History of VTE	No	265 (88)
	Unknown	9 (3)
	Yes	27 (9)
Family History of VTE	No	211 (70.1)
	Unknown	29 (9.6)
	Yes	61 (20.3)
Currently Receiving Pharmacological/Non-Pharmacological	No	71 (23.6)
	Unknown	31 (10.6)
	Yes	199 (66.1)
History of Receiving Pharmacological/Non-Pharmacological	No	189 (62.8)
	Unknown	36 (121)
	Yes	76 (25.2)

Table 2 shows that 39.2% of the studied patients reported knowing what a blood clot in their leg/DVT was and 45.5% correctly identified that a blood clot in a vein causes DVT. As for known DVT symptoms, 39.6%, 43.9%, 34.7%, and 18.8% of patients correctly identified that DVT symptoms included leg swelling, pain/tenderness, noticeable changes in skin color, and the leg feeling warm, respectively. As for risk factors for blood clots, the most common known risk factors were not moving for a long time (39.2%), high blood cholesterol (37.5%), family history of blood clots (35.5%), older age (65+) (28.9%), high blood pressure (24.9%), and surgery (22.9%). Most of the patients (66.1%) knew that walking/stretching the legs helps prevent blood clots. Almost one-third of the studied patients (29.6%) knew what a blood clot in their lung/PE was and 69.1%, 37.5%, 3.3%, and 16.6% knew that PE symptoms include shortness of breath, chest pain (may be worse with deep breaths), lightheadedness/passing out, and coughing up blood, respectively.

**Table 2:** Distribution of studied patients according to their response to knowledge items regarding VTE (No = 301) *The correct answer

Variable		No. (%)
Do you know what DVT or a blood clot in your leg is?	No	183 (60.8)
	Yes	118 (39.2)
Which of the following causes DVT?	Blood clot in the vein*	137 (45.5)
	Lack of oxygen in the vein	22 (7.3)
	A tumor in the vein	9 (3)
	Not sure	125 (41.5)
	None of the above	8 (2.7)
Which of following are signs/symptoms of DVT?	Swelling of leg*	120 (39.6)
	Itching of leg	24 (7.9)
	Pain/tenderness in leg*	133 (43.9)
	Noticeable changes in color of leg*	105 (34.7)
	The leg feels warm*	57 (18.8)
	Leg paralysis	88 (29)
	Not sure	87 (28)
Do you know what PE or a blood clot in your lung is?	No	212 (70.4)
	Yes	89 (29.6)
Which of following are signs/symptoms of PE?	Shortness of breath*	208 (69.1)
	Slow, shallow breathing	75 (24.9)
	Chest pain (may be worse with deep breath) *	113 (37.5)
			Rapid heart rate	35 (11.6)
	Light headedness/passing out *	10 (3.3)
	Pain radiating down arm	15 (5)
	Coughing up blood *	50 (16.6)
	Frequent headaches	10 (3.3)
	Other	60 (19.9)
	None of the above	25 (8.3)
	Which of the following might increase your risk of developing a blood clot?	A hospital stay *	30 (10)
Surgery *		69 (22.9)
Cancer *		18 (6)
Not moving for a long time*		118 (39.2)
Pregnancy/giving birth *		59 (19.6)
Taking estrogen-based medicines *		48 (15.9)
Family history of blood clots *		107 (35.5)
			Older age (65+) *	87 (28.9)
Too much exercise		7 (2.3)
High blood cholesterol *		113 (37.5)
Donating blood		5 (1.7)
High blood pressure *		75 (24.9)
Don’t know		10 (3.3)
Which of following helps prevent a blood clot?		Walking/stretching legs *	199 (66.1)
	Drinking plenty of fluids	134 (44.5)
	Eating lots of fiber	82 (27.2)
	Bed rest	17 (5.6)
	Washing/bathing regularly	92 (30.6)
	Don’t know	50 (16.6)
	Not sure	17 (5.6)

**Figure 1:**
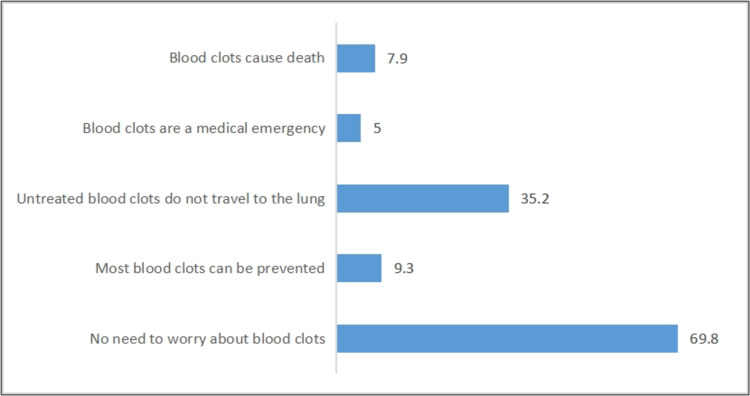
Distribution of responses (%) regarding patients’ awareness (disagree/strongly disagree responses) regarding VTE (No = 301).

Figure 1 shows that 69.8% of the participants agreed that there is a need to worry about blood clots, and 35.2% agreed that untreated blood clots could travel to the lungs. Table 3 shows that most of the participants (65.4%) agreed that the daily injections of prophylaxis are useful and they needed this injection (57.1%), while 54.9% were aware of the possible side effects. The majority (61.1%) were satisfied with the frequency of the injections and the explanation they received before taking them (47.5%). 48.5% were satisfied with the information given about thromboembolism and pulmonary disease.

**Table 3:** Distribution of responses (%) regarding patients’ perception and satisfaction with pharmacological thromboprophylaxis and information received about VTE (agree/strongly agree).

	Questions	Strongly agree No. (%)	Agree No. (%)	Neither agree nor disagree No. (%)	Disagree No. (%)	Strongly disagree No. (%)
Perception	Daily injection help my health and safety	56 (18.6%)	141 (46.8%)	78 (25.9%)	20 (6.6%)	6 (2%)
	I need these injections	38 (12.6%)	134 (44.5%)	77 (25.6%)	43 (14.3%)	9 (3%)
	Possible side effects of this treatment	42 (14%)	123 (40.9%)	96 (31.9%)	33 (11%)	7 (2.3%)
Satisfaction	The reason for injection was adequately explained	31 (10.3%)	112 (37.2%)	82 (27.2%)	61 (20.3%)	15 (5%)
	Satisﬁed with the information given about deep-vein thrombosis and pulmonary embolism	44 (14.6%)	102 (33.9%)	57 (18.9%)	79 (26.2%)	19 (6.3%)
	Acceptable time for injection	48 (15.9%)	136 (45.2%)	83 (27.6%)	32 (10.6%)	2 (0.7%)

**Figure 2:**
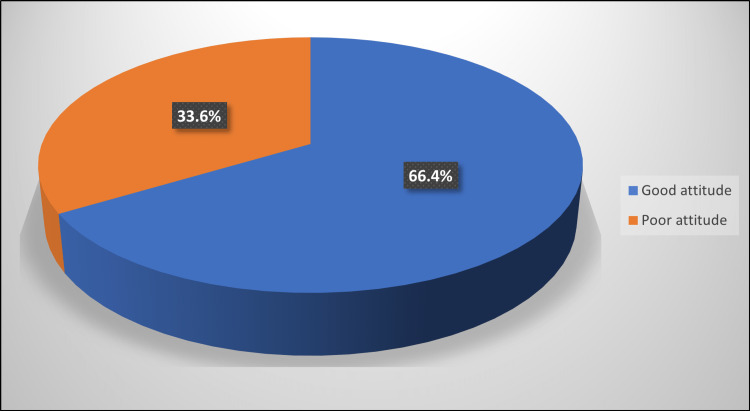
Distribution of patients (%) according to their attitude level towards VTE.

The calculated means of knowledge and attitude scores were 5.87 ± 2.59 and 6.92 ± 2.71, respectively. All the studied patients had poor knowledge of VTE, as all of them had less than an 80% level of knowledge after calculating the knowledge score. Altogether, 33.6% had a poor attitude and 66.4% had a good attitude toward VTE, respectively (Figure 2).

Table 4 shows that patients who had a higher education level and those who were currently or previously receiving pharmacological/non-pharmacological thromboprophylaxis had a significantly higher VTE knowledge score (p = < 0.05). On the other hand, a non-significant relationship was found between knowledge scores and age, gender, the reason for admission, and personal or family history of VTE (p = > 0.05).

**Table 4:** Relationship between patients’ knowledge level about VTE and their demographic characteristics, reason for admission, and clinical history (N = 301) *Kruskal Wallis test; **Mann-Whitney test

Variable		Knowledge Score (Mean ± SD)	Test	p-value
					Age Range (years)	18–30	7.09 ± 2.74	3*	0.077
31–50	7.36 ± 2.68								
51–70	6.4± 2.7								
71+	6.63 ± 2.55								
Gender	Female	6.92 ± 2.84	0.07**	0.943
	Male	6.97 ± 2.62		
Education Level	Uneducated	6.07 ± 2.77	4*	0.001
	Less than high school	6.01 ± 2.77		
	High school	7.16 ± 2.55		
	University	7.62 ± 2.5		
	Higher education	8 ± 3.26		
Reason for Admission	Chemotherapy	8 ± 0.001	2**	0.375
	Oncology(nonsurgical)	6 ± 2.25		
	Surgical	6.99 ± 2.72		
Personal History of VTE	No	7.05± 2.73	2**	0.24
	Unknown	6.11 ± 1.69		
	Yes	6.25 ± 2.59		
Family History of VTE	No	6.92 ± 2.7	2**	0.41
	Unknown	6.34 ± 2.1		
	Yes	7.26 ± 2.94		
Currently Receiving Pharmacological/non-Pharmacological	No	6.25 ± 2.62	2**	0.016
	Unknown	6.41 ± 2.01		
	Yes	7.29 ± 2.77		
History of Receiving Pharmacological/Non-Pharmacological	No	6.97 ± 2.74	2**	0.002
	Unknown	5.66 ± 1.94		
	Yes	7.51 ± 2.75		

Table 5 demonstrated that patients who were currently receiving or had previously received pharmacological/non-pharmacological thromboprophylaxis had a significantly higher level of satisfaction when they were compared to the patients who are experiencing it for the first time (p = < 0.05). On the other hand, a non-significant relationship was found between attitude level and age, gender, educational level, reason for admission, and personal or family history of VTE (p=> 0.05).

**Table 5:** Relationship between patients’ attitude toward VTE and their demographic characteristics, reason for admission, and clinical history (N = 301)

Variable		Attitude level		χ2	p-value
		Poor No. (%)	Good No. (%)		
						Age Range (years)	18–30	40 (72.7)	15 (27.3)	3.41	0.332
31–50	76 (60.8)	49 (39.2)									
51–70	60 (70.6)	256(29.4)									
71+	24 (66.7)	12 (33.3)									
Gender	Female	77 (67)	38 (33)	0.02	0.833
	Male	123 (66.1)	63 (33.9)		
Education Level	Uneducated	32 (80)	8 (20)	7.42	0.115
	Less than high school	44 (69.8)	19 (30.2)		
						High school	60 (67.4)	29 (32.6)
	University	61 (59.8)	41 (40.2)					
	Higher education	3 (42.9)	4 (57.1)		
	Reason for Admission	Chemotherapy	1 (100)			0 (0.0)	4.1	0.128
		Oncology (nonsurgical)	11 (91.7)			1 (8.3)		
Surgical		188(65.3)	100(34.7)					
Personal History of VTE	No	175 (66)	90 (34)	0.53	0.764
	Unknown	7 (77.8)	2 (22.2)		
	Yes	18 (66.7)	9 (33.3)		
Family History of VTE	No	136(64.5)	75 (35.5)	2.54	0.28
	Unknown	23 (79.3)	6 (20.7)		
	Yes	41 (67.2)	20 (32.8)		
Currently Receiving Pharmacological/Non-Pharmacological	No	58 (81.7)	13 (18.3)	17.55	<0.001
	Unknown	26 (83.9)	5 (16.1)		
	Yes	116(58.3)	83 (41.7)		
History of Receiving Pharmacological/Non-Pharmacological	No	130(68.8)	59 (31.2)	6.97	0.033
	Unknown	28 (77.8)	8 (22.2)		
	Yes	42 (55.3)	34 (44.7)		

**Figure 3:**
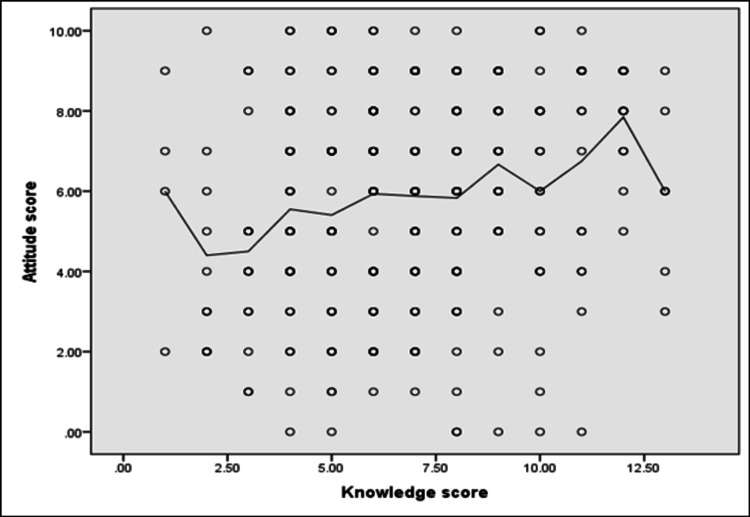
Spearman’s correlation analysis between patients’ knowledge and attitude scores Note: (r = 0.21, p = < 0.001)

Figure 3 illustrates that a highly significant positive correlation was found between patients’ knowledge and attitude scores (r = 0.21, p = < 0.001).

## Discussion

The main findings of this study were that hospitalized patients in the surgical ward in Al-Noor Hospital have poor awareness of DVT and PE (39% and 30%, respectively). Correspondingly, they demonstrate a lack of understanding of the signs and symptoms of DVT and PE. These findings are consistent with previous similar studies done among the general population in different countries [10,11]. Another two cross-sectional surveys conducted among hospitalized patients in Jordan and Saudi Arabia found that hospitalized patients have poor knowledge of and awareness of VTE [8,9]. Moreover, the lack of awareness of DVT and PE is not limited to specific types of patients, as there have been studies conducted among, for example, cancer patients, pregnant women, and postpartum women showing similar results [12,13].

Our findings show that patients have insufficient knowledge about the risk factors of VTE and prevention measures. This is unfortunate, as hospitalized patients should actively participate in VTE prevention. The most commonly reported risk factor of VTE among the study participants was immobility, which corroborates with findings reported in a previous study [7]. This result reflects the attempts of healthcare providers to encourage hospitalized patients to move as much as possible.

However, participants were less familiar with other risk factors, such as cancer, pregnancy, and surgery, even though this study was conducted among patients hospitalized in the surgical ward. These results could be attributed in part due to health care providers’ tendency to pay more attention to immobility than any other risk factors when educating their patients.

Our study showed that the participants were more likely to agree that blood clots could be prevented (61%). This result is consistent with an earlier study, in which 55% of participants agreed that blood clots could be prevented [9]. However, only 28% of our patients agreed that untreated blood clots could spread to the lungs. This result is in agreement with a study by Almodaimegh et al. which found that only 37% of their participants knew that untreated blood clots could spread to the lungs [8]. These findings indicated that the relationship between DVT and PE is unknown, probably because of the pathophysiological nature of PE.

Regarding the perception of thromboprophylaxis, nearly half of the studied patients received information about DVT and PE. Such findings likely explain the current study participants’ lack of awareness of VTE and its manifestations, highlighting the need for education to improve the patient's knowledge of VTE and thromboprophylaxis.

The current study also investigated factors that affect the awareness of VTE. A significant relationship was found between the patients’ knowledge about VTE and higher educational levels. A randomized controlled study showed that after nurses provided educational programs to postpartum women, awareness and knowledge regarding VTE increased and improved dramatically, from 8% to 87% [14]. This finding suggests that encouraging patients to participate in educational programs on VTE and its symptoms can reduce the incidence of hospital-acquired VTE.

DVT and PE are common preventable causes of morbidity and mortality in hospitalized patients and many healthcare systems are trying to decrease their event rates. Raising the level of awareness among the general population, especially among hospitalized and high-risk patients, could have a significant impact on patient compliance regarding prophylaxes, early immobilization, and self-reported signs and symptoms. A randomized clinical trial conducted among hospitalized patients showed a significant increase in the level of awareness and knowledge about DVT and PE after the implementation of educational programs [14].

Study limitations

Our study has several limitations. First, the closed-ended questions accompanied by a provided list of options may help the participants make a guess rather than responding based on their knowledge. Additionally, the recommended sample size was achieved, but increasing the sample size may allow for a more reliable conclusion. Also, the study was conducted at one hospital, so the findings may not apply to all hospitals in Saudi Arabia.

## Conclusions

This study has revealed a lack of awareness about DVT and PE among hospitalized patients. Patients were unaware of the disease, its manifestations, and its risk factors. The findings of this study should encourage health care providers to educate patients and public health organizations about DVT, PE, their risk factors, signs and symptoms, preventive measures, and the role of thromboprophylaxis in reducing the risk of VTE.

## References

[1] Schulman S, Ageno W, Konstantinides SV (2017). Venous thromboembolism: Past, present and future. Thromb Haemost.

[2] Heit JA, Spencer FA, White RH (2016). The epidemiology of venous thromboembolism. J Thromb Thrombolysis.

[3] Al-Hameed F, Al-Dorzi HM, Shamy A (2015). The Saudi clinical practice guideline for the diagnosis of the first deep venous thrombosis of the lower extremity. Ann Thorac Med.

[4] Serhal M, Barnes GD (2019). Venous thromboembolism: A clinician update. Vasc Med.

[5] Nicholson M, Chan N, Bhagirath V, Ginsberg J (2020). Prevention of venous thromboembolism in 2020 and beyond. J Clin Med.

[6] Wendelboe AM, Raskob GE (2016). Global burden of thrombosis: epidemiologic aspects. Circ Res.

[7] Le Sage S, McGee M, Emed JD (2008). Knowledge of venous thromboembolism (VTE) prevention among hospitalized patients. J Vasc Nurs.

[8] Almodaimegh H, Alfehaid L, Alsuhebany N, Bustami R, Alharbi S, Alkatheri A, Albekairy A (2017). Awareness of venous thromboembolism and thromboprophylaxis among hospitalized patients: a cross-sectional study. Thromb J.

[9] Jarab AS, Azzam SA, Badaineh R, Mukattash TL, Bsoul R (2020). Awareness and perception of thromboembolism and thrombo-prophylaxis among hospitalized patients in Jordan. Curr Clin Pharmacol.

[10] Alyahya KI, Alangari FS, Saja DK, Alothaim LO, Albugami SJ, Sama O (2020). Public awareness of venous thromboembolism in Riyadh, Saudi Arabia. International Journal of Advanced and Applied Sciences.

[11] Okoye H, Nwagha T, Ezigbo E (2021). Low awareness of venous thromboembolism among the general population: a call for increased public enlightenment programs. J Prev Med Hyg.

[12] Kim ES, Kim HY (2019). Knowledge, awareness and risk of occurrence of venous thromboembolism of perinatal women. Korean J Women Health Nurs.

[13] Sousou T, Khorana AA (2010). Cancer patients and awareness of venous thromboembolism. Cancer Invest.

[14] Youness EM, Hassen GH, Youness HM, Azer SZ (2016). Effect of educational nursing program on reducing the incidence of venous thromboembolism among postpartum women. IOSR Journal of Nursing and Health Science.

